# A machine learning-based predictive model for biliary stricture attributable to malignant tumors: a dual-center retrospective study

**DOI:** 10.3389/fonc.2024.1406512

**Published:** 2024-07-29

**Authors:** Qifan Yang, Lu Nie, Jian Xu, Hua Li, Xin Zhu, Mingwei Wei, Jun Yao

**Affiliations:** ^1^ Department of Gastroenterology, The Affiliated People’s Hospital of Jiangsu University, Zhenjiang, Jiangsu, China; ^2^ Department of Intervention Vascular, Wujin Hospital Affiliated with Jiangsu University, Changzhou, China; ^3^ Department of General Surgery, Affiliated Hospital of Youjiang Medical University for Nationalities, Baise, China; ^4^ Key Laboratory of Tumor Molecular Pathology of Baise, Baise, China

**Keywords:** malignant tumors, biliary stricture, risk factors, machine learning, predictive model

## Abstract

**Background:**

Biliary stricture caused by malignant tumors is known as Malignant Biliary Stricture (MBS). MBS is challenging to differentiate clinically, and accurate diagnosis is crucial for patient prognosis and treatment. This study aims to identify the risk factors for malignancy in all patients diagnosed with biliary stricture by Endoscopic Retrograde Cholangiopancreatography (ERCP), and to develop an effective clinical predictive model to enhance diagnostic outcomes.

**Methodology:**

Through a retrospective study, data from 398 patients diagnosed with biliary stricture using ERCP between January 2019 and January 2023 at two institutions: the First People’s Hospital affiliated with Jiangsu University and the Second People’s Hospital affiliated with Soochow University. The study began with a preliminary screening of risk factors using univariate regression. Lasso regression was then applied for feature selection. The dataset was divided into a training set and a validation set in an 8:2 ratio. We analyzed the selected features using seven machine learning algorithms. The best model was selected based on the Area Under the Receiver Operating Characteristic (ROC) Curve (AUROC) and other evaluation indicators. We further evaluated the model’s accuracy using calibration curves and confusion matrices. Additionally, we used the SHAP method for interpretability and visualization of the model’s predictions.

**Results:**

RF model is the best model, achieved an AUROC of 0.988. Shap result indicate that age, stricture location, stricture length, carbohydrate antigen 199 (CA199), total bilirubin (TBil), alkaline phosphatase (ALP), (Direct Bilirubin) DBil/TBil, and CA199/C-Reactive Protein (CRP) were risk factors for MBS, and the CRP is a protective factor.

**Conclusion:**

The model’s effectiveness and stability were confirmed, accurately identifying high-risk patients to guide clinical decisions and improve patient prognosis.

## 1 Introduction

Biliary stricture caused by malignant tumors is known as Malignant Biliary Stricture (MBS). Among patients diagnosed with biliary stricture through Endoscopic Retrograde Cholangiopancreatography (ERCP), distinguishing between MBS and Benign Biliary Stricture (BBS) presents a significant clinical challenge (1, 2). MBS are commonly associated with cancers like cholangiocarcinoma, pancreatic cancer, gallbladder cancer, and metastases from other systemic cancers. Conversely, BBS frequently result from a multitude of complex factors, such as primary sclerosing cholangitis (PSC), both acute and chronic pancreatitis, cholangiolithiasis, injuries to the biliary system, and infections (3, 4). Therefore, early and precise diagnosis of MBS, or the development of a reliable diagnostic approach for cases with a high suspicion of MBS, is crucial for enhancing patient outcomes and directing treatment plans. Distinguishing between MBS and BBS continues to be a significant clinical challenge. Due to the specificity of the biliary tract structure and the complexity of the aetiology of malignant bile duct stenosis, the sensitivity of commonly used diagnostic methods is low. ERCP-guided biopsy and brushing are the most commonly used diagnostic methods in clinical practice, and its sensitivity is low, even when combined with the circulating tumor biomarker carbohydrate antigen 19–9 (CA199), which has a sensitivity between 14% and 60% (5). The combined diagnostic accuracy of biliary brushing, intraductal biopsy, and fluorescence *in situ* hybridization (FISH) is approximately 82% (6). Ultrasound endoscopy (EU) is also one of the commonly used methods for clinical diagnosis of MBS, including ultrasound endoscopic aspiration (EUA) and ultrasound endoscopic puncture biopsy (EUB), and its sensitivity is greatly improved compared to that of ERCP biopsy and brushing, which is about 80% (7). However, the complexity and associated risks of EUS procedures constrain their extensive application. Additionally, concerns about the potential for tumor dissemination during EUS punctures necessitate cautious utilization of this technique (5). And new technologies, such as transoral cholangioscopy and probe-based confocal laser microendoscopy, require the introduction of high-precision instruments, wider clinical validation and a long learning curve that are difficult to roll out in the short term. Furthermore, these resources are not readily available in most hospitals, and there has been minimal improvement in the diagnostic aspect of MBS in most hospitals.

Due to the low detection rate and the complex nature of MBS etiology, patients often need multiple ERCP examinations for diagnosis. This can lead to delayed treatment, adversely affecting timely patient care (8). ERCP and similar endoscopic examinations, as invasive procedures, entail certain risks. Moreover, the need for repeated examinations can result in reduced patient compliance. Consequently, deciding whether to subject a patient to repeated ERCP and additional invasive tests presents a considerable challenge for clinicians. This scenario necessitates a careful equilibrium between the necessity for precise diagnosis and the potential risks and patient compliance challenges associated with these procedures. Inaccurate diagnoses of MBS can result in unnecessary multiple ERCP or even surgical interventions for patients with BBS, profoundly impacting their quality of life. Early diagnosis and identification of high-risk patients are essential for establishing treatment strategies and enhancing outcomes for individuals with MBS. Current research is mainly directed at identifying patients whose endoscopic tissue samples lack tumor tissues but who clinically exhibit symptoms indicative of MBS. However, the clinical evaluation to determine if a patient is at high risk is susceptible to missed diagnoses. This makes the secondary screening process for high-risk patients limited (9). Our study extends to a wider scope, extracting characteristics of patients with MBS from all cases of biliary strictures.

We have developed a new predictive model based on machine learning algorithms to explore the risk factors most closely associated with MBS. This allows clinicians to accurately identify high-risk patients without the need for biopsy, providing new evidence to support clinical decisions and the formulation of related diagnostic and treatment strategies.

## 2 Materials and methods

### 2.1 Study population and data collection

This is a dual-center retrospective study that collected data from all patients diagnosed with biliary stricture through ERCP from January 2019 to January 2023 at the First People’s Hospital affiliated with Jiangsu University and the Second People’s Hospital affiliated with Suzhou University. A 12-month follow-up was conducted for all patients to confirm their diagnoses.

The inclusion criteria for the study were: 1) age range between 18–90 years; 2) biliary stricture diagnosis established via ERCP; 3) confirmation of diagnosis during hospitalization through surgical procedures and endoscopic pathology; 4) unclear diagnosis during hospitalization but confirmed within a year of follow-up or due to death attributable to malignant biliary stricture.

The exclusion criteria encompassed: 1) severe cardiopulmonary dysfunction; 2) death from other diseases or accidents during hospitalization without a definitive diagnosis; 3) death from other diagnosed diseases or accidents during the follow-up period without a clear diagnosis; 4) significant loss or complete absence of data.

The study involved the collection and documentation of initial hospitalization data and patient histories. This included demographics (age, gender), stricture characteristics location(the common bile duct (CBD), common hepatic duct (CHD) and above), stricture length, carbohydrate antigen 199 (CA199), direct bilirubin (DBil), total bilirubin (TBil), gamma-glutamyl transferase (GGT), alkaline phosphatase (ALP), aspartate aminotransferase (AST), C-reactive protein (CRP), alanine aminotransferase (ALT). Patient lifestyle factors, including smoking status, alcohol consumption, and the presence of hypertension or diabetes, were also recorded. Additionally, three extra indicators were considered, based on previous research: DBil/TBil, CA199/CRP, and CA199/TBil (10–12).

### 2.2 Statistical methods

Categorical variables were reported as the count (percentage) and evaluated using the chi-square (χ2) test. The Kolmogorov-Smirnov test indicated that the quantitative data were not normally distributed, and thus were presented as median (lower and upper quartiles), with the Mann-Whitney U test used for the comparison of two independent samples. Firstly, univariate analysis was conducted using binary logistic regression on the data after cleaning, selecting variables significant at p<0.05. Subsequently, the data underwent Z-score standardization to ensure that variables of different scales do not improperly influence the model due to their range of values. Lasso regression with the optimal alpha value was then utilized for feature selection. The dataset was divided into a training set and a validation set in an 8:2 ratio. The training dataset was modeled using Random Forest (RF), Support Vector Machine (SVM), Logistic Regression (LR), AdaBoost, Neural Networks (NN), K-Nearest Neighbors (KNN), and Decision Trees (DT) machine learning algorithms, and a 10-fold cross-validation was conducted to improve model accuracy, followed by validation with the validation set. Finally, the constructed models were evaluated using the area under the receiver operating characteristic (AUROC), confusion matrix, and evaluation indicators to select the best model. The model’s visualization was achieved through the Shapley Additive explanations (SHAP) method, highlighting the contributions of important features and the best indicators in the optimal model. Data analysis for this study was performed using Python (version 3.11) and SPSS (version 27.0).

## 3 Results

### 3.1 Etiological investigation of MBS

**Figure 1:**
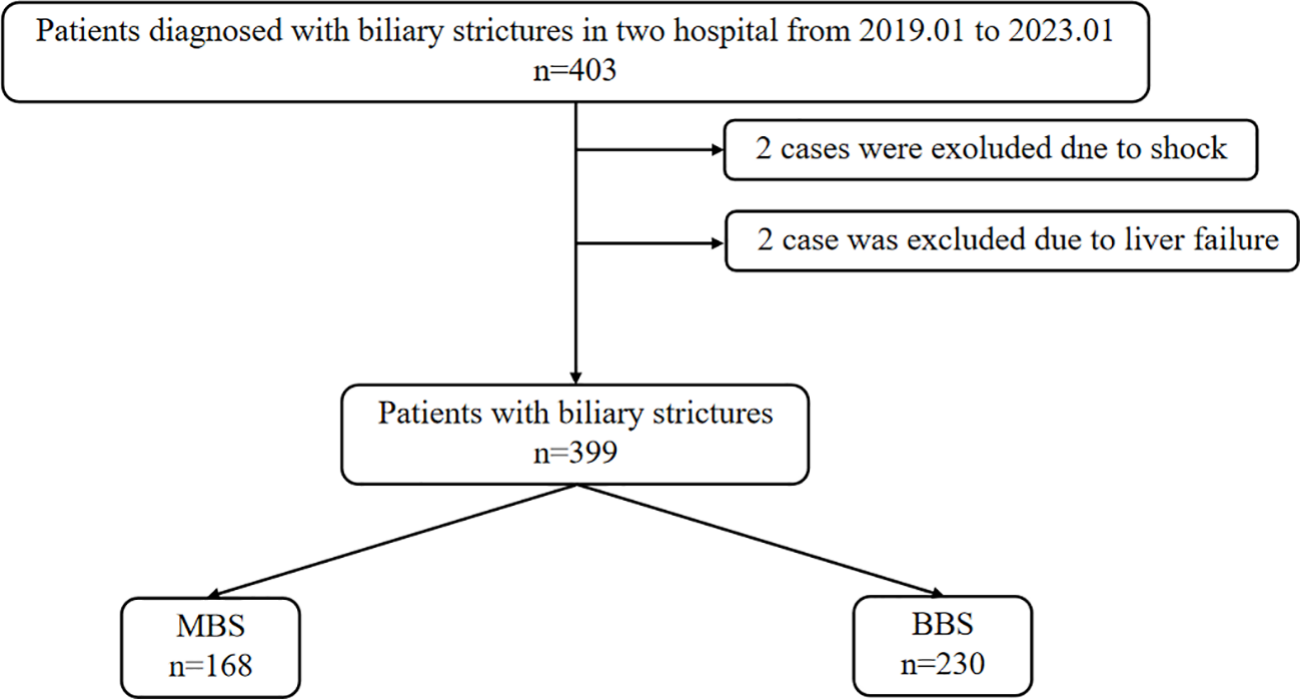
Patient screening and grouping flowchart. *MBS,* malignant biliary stricture; *BBS*, benign biliary stricture.

This study aggregated data from 284 patients at the First People’s Hospital of Zhenjiang and 119 patients from the Second People’s Hospital affiliated with Soochow University, totaling 403 cases. Upon admission, two patients were in shock, two patients succumbed to liver failure during hospitalization. Following the inclusion and exclusion criteria, 398 patients were considered for the study, comprising 168 cases of MBS and 230 cases of BBS (**Figure 1**).

Within the MBS category, the majority were pancreatic cancer cases, numbering 78 (46.4%), cholangiocarcinoma cases stood at 53 (31.5%), both duodenal papillary and gallbladder cancers were recorded at 10 cases each (6.0%), liver cancer had 6 cases (3.6%), and metastatic cancer also had 10 cases (6.0%). For BBS, the prevalent cause was stones, with 111 cases (48.3%) of simple bile duct stones, 20 cases (8.7%) of cholangitis, another 20 cases (8.7%) combining bile duct stones and cholangitis, and 34 cases (14.8%) were due to gallbladder stones with cholecystitis. Pancreatitis was a significant contributor to BBS, with acute pancreatitis at 18 cases (7.8%), chronic pancreatitis at 2 cases (0.8%), and autoimmune pancreatitis at 4 cases (1.7%). The study also recorded 4 cases (1.7%) of primary sclerosing cholangitis and 18 cases (7.8%) of postoperative bile duct stricture.

### 3.2 The baseline characteristics and univariate analysis of biliary stricture

The study detailed demographic characteristics, laboratory data, and univariate analysis of patients with biliary strictures in **Table 1**. In the univariate LR analysis, age, location of stricture, length of stricture, CA199, TBil, DBil, ALP, GGT, DBil/TBil, and CA199/CRP were identified as risk factors for MBS (P>0.05). Based on the results of the correlation diagnostics, ALP and GGT were found to be highly correlated (Pearson correlation coefficient: 0.59), leading to the decision to include ALP in the model.

**Table 1:** The baseline characteristics and univariate analysis of biliary stricture.

Variables	Total (n =398)	MBS (n =168)	BBS (n =230)	*Z/*χ^2^	*OR*	*95%CI*	*P* value
Sex				0.035	1.039	0.695–1.555	
Male	230 (57.8%)	98 (58.3%)	132 (57.4%)				0.851
Female	168 (42.2%)	70 (41.7%)	98 (42.6%)				
Age(years)	70 (58, 77)	71 (65, 76)	9 (54, 77)	2.556	1.026	1.011–1.042	0.001
Stricture Part				10.976	2.579	1.453–4.575	
CBD	340 (85.4%)	132 (78.6%)	208 (90.4%)				0.001
CHD and above	58 (14.6%)	36 (21.4%)	22 (9.6%)				
Stricture length(mm)	10 (5, 21)	20 (13, 26)	6(4, 11)	10.5	1.113	1.086–1.141	<0.001
CA199(u/L)	112.81 (24.38,421.42)	305.80 (73.17,1000)	58.00 (15.61, 211.38)	7.384	1.002	1.001–1.002	<0.001
TBil(umol/L)	73.67 (28.55,150.50)	140.20 (87.15,220.10)	38.75 (18.05,83.15)	10.83	1.013	1.010–1.016	<0.001
AST(u/L)	94 (47,197)	114.50 (68.25,201.25)	74.50 (32.25,187.50)	3.555	1.000	0.999–1.001	0.735
ALT(u/L)	128 (58,255.25)	149 (84,259)	116 (39,251.5)	2.381	1.000	0.999–1.001	0.968
GGT(u/L)	364 (182.75,742.75)	491 (297.5,910.75)	303 (116.25,510.75)	6.139	1.001	1.001–1.002	<0.001
ALP(u/L)	243 (145.75,415)	379.5 (244,624.25)	177.5 (115,273.75)	9.498	1.003	1.002–1.004	<0.001
CRP(mg/L)	10.37 (4.71,45.43)	10.39 (4.71,36.22)	9.8 (4.78,53.16)	-0.449	0.995	0.991–0.999	0.030
Smoking history				0.296	0.855	0.785–1.505	
No	339 (85.2%)	145 (86.3%)	194 (84.3%)				0.587
Yes	59 (14.8%)	23 (13.7%)	36 (15.7%)				
Drinking history				0.593	0.769	0.394–1.502	
No	357 (89.7%)	153 (91.1%)	204 (88.7%)				0.442
Yes	41 (10.3%)	15 (8.9%)	26 (11.3%)				
Hypertension				1.352	1.269	0.849–1.897	
No	229 (57.5%)	91 (54.2%)	138 (60.0%)				0.245
Yes	169 (42.5%)	77 (45.8%)	92 (40.0%)				
Diabetes				0.549	1.202	0.739–1.955	
No	315 (79.1%)	130 (77.4%)	185 (80.4%)				0.459
Yes	83 (20.9%)	38 (22.6%)	45 (19.6%)				
DBil/TBil	71(54,78)	76.5(72,80)	62(44,74.75)	8.446	1.064	1.047–1.081	<0.001
CA199/CRP	9.72(1.98,37)	24.05(5.79,96.48)	4.95(1.34,19.44)	6.688	0.012	1.007–1.017	<0.001
CA199/TBil	2(0.54,5.33)	2.58(0.57,6.72)	1.6(0.53,4.43)	1.651	1.012	0.983–1.042	0.430

MBS, malignant biliary stricture; BBS, benign biliary stricture; CBD, common bile duct; CHD, common hepatic duct; CA199, carbohydrate antigen 199; TBil, total bilirubin; DBil, direct bilirubin; AST, aspartate aminotransferase; ALT, alanine aminotransferase; GGT, gamma-glutamyl transferase; ALP, alkaline phosphatase; CRP, C-reactive protein.

### 3.3 Lasso regression to select feature variables for MBS

**Figure 2:**
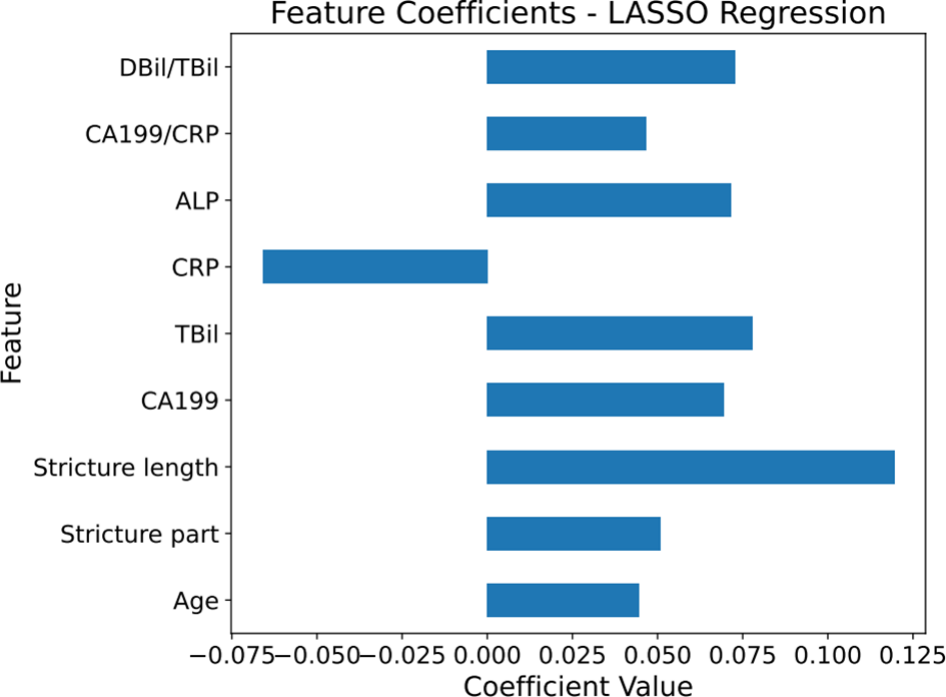
Horizontal bar chart of feature coefficients from Lasso regression. The histogram displays the coefficient values of different features when using LASSO regression to predict malignant biliary stricture (MBS).The magnitude of the coefficient values represents the weight of the features in the predictive model, with positive values corresponding to features that increase the likelihood of predicting malignancy in MBS, and negative values indicating features that decrease this likelihood. The numerical values on the horizontal axis show the magnitude of this influence, reflecting their importance. The blue bars in the graph represent the coefficient values, with longer bars indicating a greater influence of the feature on the model. *CA199*, carbohydrate antigen 199; *TBil*, total bilirubin; *DBil*, direct bilirubin; *ALP*, alkaline phosphatase; *CRP*, C-reactive *protein*.

Using LassoCV, we conducted cross-validation across a range of candidate regularization parameters, ultimately identifying an optimal alpha value of 0.01916. With this parameter, Lasso regression revealed that age, stricture location, stricture length, CA199, TBil, ALP, GGT, DBil/TBil, and CA199/CRP significantly contribute to predicting MBS. These factors were visually demonstrated in a histogram (**Figure 2**).

### 3.4 Construction and validation of the MBS prediction model

Grid search (GridSearchCV) was employed alongside ten-fold cross-validation to ensure robustness in the parameter tuning process. Subsequently, the ROC curve for the training set was plotted. Considering AUROC as the primary criterion and precision, accuracy, recall, and F1 score as secondary considerations (**Table 2**), we identified that the Random Forest model exhibited superior performance.

**Table 2:** Performance indicators of the machine learning model.

Model	Accuracy	Precision	Recall	F1-Score	AUC
RF	0.888	0.892	0.888	0.886	0.988
SVM	0.900	0.903	0.900	0.899	0.935
LR	0.888	0.898	0.888	0.886	0.883
NN	0.888	0.889	0.888	0.887	0.931
KNN	0.850	0.850	0.850	0.850	0.918
DT	0.813	0.815	0.813	0.811	0.969
AdaBoost	0.875	0.878	0.875	0.874	0.943

RF, Random Forest, SVM, Support Vector Machine, LR, Logistic Regression, AdaBoost, NN, Neural Networks, KNN, K-Nearest Neighbors, DT, Decision Trees.

**Figure 3:**
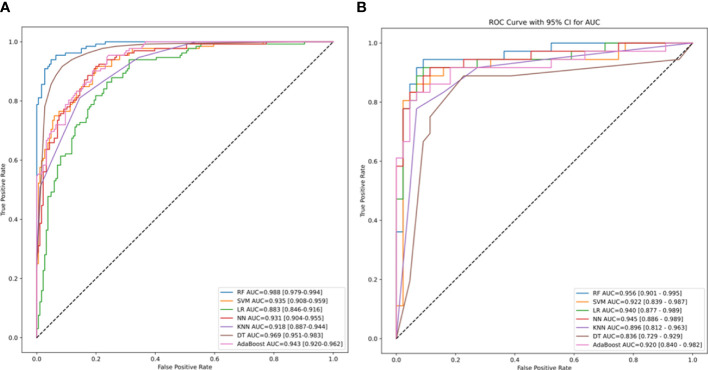
The area under the receiver operating characteristic (ROC) curve (AUROC) of machine-learning models. The AUROC and its 95% confidence intervals for the training set RF, SVM, LR, DT, KNN, NN, AdaBoost models, representing the sensitivity plotted against the specificity for individual risk factors of MBS. The dotted line represents the 50% AUC value. **(B)** The AUC and its 95% confidence intervals for the ROC curves generated by various machine learning models of the validation set. RF, Random Forest; SVM, Support Vector Machine; LR, Logistic Regression; AdaBoost, NN, Neural Networks; KNN, K-Nearest Neighbors; and DT, Decision Trees.

In the test set, RF model achieved an AUROC of 0.988, with a 95% confidence interval (CI) of 0.979–0.994(**Figure 3**), demonstrating excellent predictive performance and stability. The test set AUC was 0.956. For other machine learning models: SVM: 0.935 (95% CI: 0.908–0.959).LR 0.883 (95% CI: 0.846–0.959). NN 0.931 (95% CI: 0.904–0.955). KNN 0.918 (95% CI: 0.887–0.944). DT: 0.969 (95% CI: 0.951–0.983). The AdaBoost model had an AUC score of 0.943 (95% CI: 0.920–0.962). The AUCs for the validation set were as follows: RF - 0.922, SVM - 0.940, LR - 0.945, NN - 0.996, KNN - 0.836, and DT - 0.920.

**Figure 4:**
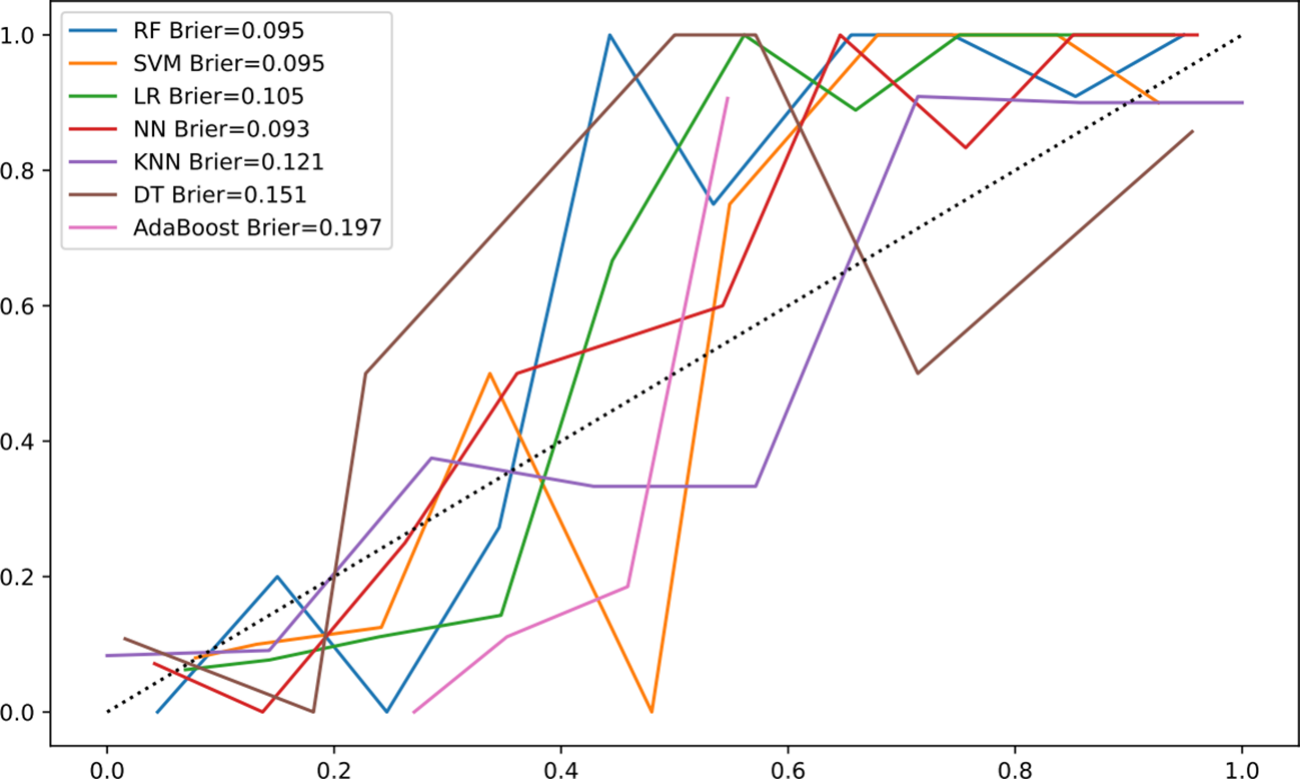
Calibration curve of machine-learning models. Calibration curve comparing the predicted probabilities of malignant biliary stricture (MBS) from the RF model with the observed probabilities. The closer the decision curve is to the ideal (large shadow) line, the better the performance of the model. The horizontal axis represents the predicted probability of MBS occurrence, while the vertical axis represents the actual observed probability of MBS occurrence.

The Brier score for the calibration curve of RF algorithm was 0.095 (**Figure 4**), indicating the model has good stability.

**Figure 5:**
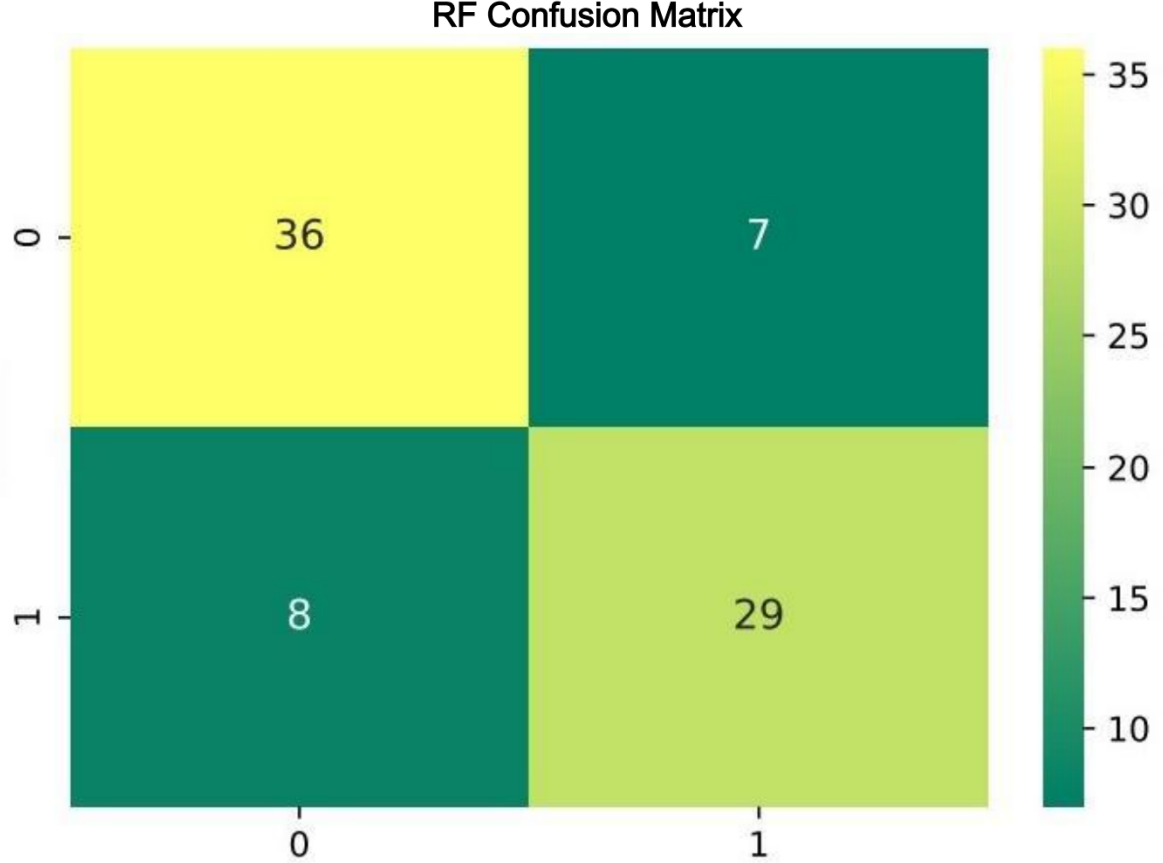
Confusion matrix for the Random Forest classifier. The rows of the matrix represent the true labels, and the columns represent the predicted labels by the model. The top-left and bottom-right cells display the counts of true negatives and true positives, respectively. The top-right and bottom-left cells show the counts of false positives and false negatives, respectively. The depth of color in the matrix cells indicates the number of samples, with darker colors representing higher counts, as illustrated by the color bar on the right side.

The confusion matrix for RF algorithm demonstrated excellent resolution (**Figure 5**).

**Figure 6:**
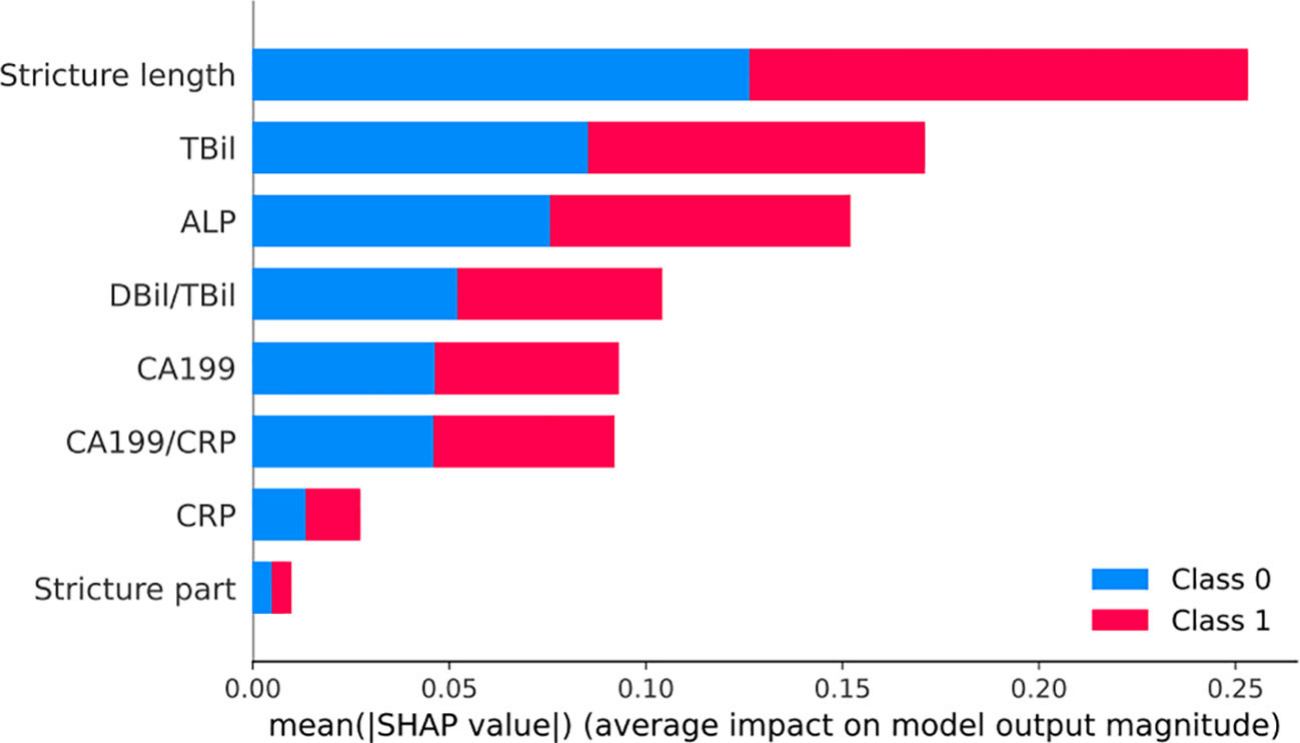
SHAP visualization chart for the Random Forest model. Bar chart of mean SHAP values demonstrating the influence of various clinical features on a model predicting benign (Class 0, blue) and malignant (Class 1, red) biliary strictures. Longer bars indicate stronger predictive impact, with Stricture length and TBil being the most influential for Class 1.

According to the SHAP results, age, stricture location, stricture length, CA199, TBil, ALP, CRP, TBil/DBil, and CA199/CRP were identified as risk factors for MBS diagnosis. The ranking of these factors is illustrated in the **Figure 6**.

## 4 Discussion

In our retrospective study, we collected data from 398 patients diagnosed with biliary stricture through ERCP at two centers. Among these, 168 cases were diagnosed as MBS. Notably, 12 patients initially of unknown etiology during their first hospitalization were later diagnosed with MBS either during follow-up or in subsequent hospital admissions. This underscores the importance of accurately identifying patients at high risk for MBS.

In this study, we aimed to develop an optimal model for diagnosing biliary strictures attributed to malignancies. Our primary metric for evaluation was the AUROC, chosen for its comprehensive ability to assess the model’s differentiation between cases and non-cases. RF model outperformed others, achieving an AUROC of 0.988, significantly higher than its counterparts. Additionally, the RF model excelled in accuracy, precision, recall, and F1 score. Consequently, our findings indicate that the Random Forest model is the most effective for diagnosing malignancy-induced biliary strictures.

Our research identified the most critical risk factors for diagnosing MBS as age, stricture location, stricture length, CA199, TBil, ALP, CRP, DBil/TBil, and CA199/CRP. Age, a factor extensively studied in relation to various cancers, is particularly significant as a risk factor for pancreatic cancer, which is closely associated with MBS (13, 14). Studies by Rustagi et al. (15) indicate that patients over the age of 60 are more susceptible to biliary or gallbladder cancer. Both of these are important causes of MBS. Our research supports the view that age is an independent risk factor for MBS. Furthermore, longer strictures are indicative of a higher risk of malignancy. This may be due to malignant strictures causing more complete and long-lasting biliary obstruction, in contrast to benign causes such as biliary stones, which can move and consequently cause varying degrees of obstruction (16). Current research on the correlation between stricture length and MBS is limited. The study by Zu et al. (17) reveals that the length of strictures in MBS is indicative of patient survival and prognosis. This finding underscores the significance of stricture length as a critical factor in assessing the severity and potential outcomes of MBS. Our study further validates its predictive value. Our study also discovered that strictures in the common hepatic duct or above have a higher likelihood of indicating MBS. This finding is likely related to the extensive distribution of bile ducts within the liver, which results in a higher incidence of biliary cancer occurring at the upper positions of the bile ducts. This correlation highlights the importance of considering the location of the stricture in diagnosing and assessing the risk of MBS, emphasizing the need for careful evaluation in these areas.

CA199 is commonly utilized in diagnosing and monitoring oncologic biliary and pancreatic diseases, particularly significant in cholangiocarcinoma and pancreatic adenocarcinoma (18).. CA199 exhibits high sensitivity but low specificity in diagnosing MBS, as its levels can also be notably elevated in conditions like cholangitis and cholelithiasis. This elevation occurs due to mechanical obstruction in the bile duct, leading to CA199, secreted by the biliary or pancreatic epithelium into the bile, leaking into the bloodstream. Additionally, secondary inflammation can prompt the normal biliary or pancreatic duct epithelium to produce more CA199, further increasing serum CA199 levels (11). In a study involving 75 individuals, Márquez et al. (19) concluded that a serum CA199 level exceeding 85.4 U/ml is strongly associated with neoplastic biliary strictures. In our research, the best AUROC for CA199 was observed at 168.9 U/ml. This finding is consistent with Márquez’s conclusions and indicates that higher thresholds might be necessary to enhance diagnostic accuracy. Marrelli et al. (20) contended that persistently high CA199 levels, specifically measurements above 90 U/mL, following the relief of biliary obstruction, are strongly indicative of MBS. Despite the limitations in specificity, CA199 continues to be a key diagnostic criterion for MBS. In this study, CRP was identified as a protective factor for MBS, indicating that CRP has promising potential in distinguishing between BBS and MBS. This may be attributed to the observation that CRP is markedly elevated in the context of inflammation, while exhibiting a relatively minor elevation in the setting of MBS. Greca et al. (10) suggested that the CA199/CRP might be a more effective diagnostic indicator for obstructive jaundice. Meanwhile, Liu et al. (11) suggested the use of TBil to mitigate the influence of inflammation on the diagnosis of MBS. In our study, these adjustments to the diagnostic criteria, such as utilizing the CA199/CRP or incorporating TBil, did not yield significant results (P>0.05). This outcome may be attributed to difference in our study’s inclusion criteria. Subsequently, we will adjust the inclusion criteria to explore or verify which indicators are more meaningful for calibrating CA199. We can enhance the accuracy and reliability of CA199 as a diagnostic and prognostic tool in clinical practice.

TBil is widely used to assess liver function and can help predict the malignancy of biliary strictures. Garcea et al. (21) in a study of 1026 patients with obstructive jaundice, found that a bilirubin level exceeding 100 umol/L had the highest sensitivity (71.9%) and specificity (86.9%) for predicting malignancy. When the threshold was increased to 400 umol/L, the specificity rose to 100%. Additionally, a decrease in TBil levels is associated with a better prognosis for patients with MBS (22). Patients with MBS typically exhibit higher bilirubin levels due to tumor progression, making elevated bilirubin an important positive predictor for malignancy. Our study also corroborated this viewpoint. The reason for lower bilirubin levels in benign disease patients might be due to earlier medical consultation prompted by pain or sepsis associated with most cases of BBS, leading to not very significant increases in TBil at the time of consultation. In chronic disease cases, where the obstruction is mild, a rapid increase in TBil is generally not observed. However, MBS often does not present significant early symptoms, leading to delayed medical consultation. As the tumor progresses, more apparent clinical symptoms emerge in a short period, leading to significant accumulation and elevation of TBil in the body. There are no previous studies on DBil/TBil in MBS, but its severity in hepatobiliary diseases has been confirmed. Lee (23) concluded that DBil/TBil is valuable in assessing both disease severity and prognosis in patients with liver disease. A study by Ma (12) showed that DBil/TBil is an independent risk factor for 90-day mortality in liver failure. This is similar to the results of the present study that DBil/TBil is an important risk factor for MBS.

ALP primarily synthesized in the liver and excreted through the biliary system. In cases of obstructive jaundice, ALP levels significantly increase, often surpassing the increase seen in AST (16). This notable elevation of ALP is a key indicator in diagnosing and assessing liver and biliary system conditions. In obstructive biliary stone disease, AST levels can rise to match or even exceed ALP levels during peak jaundice and pain episodes. This differential increase underscores the diagnostic significance of ALP in identifying MBS, as its elevated levels are more specifically associated with MBS than with BBS. Thomasset et al. (24) also indicates that in patients with biliary obstruction, an elevated ALP is more likely to suggest MBS. Our study supports a similar conclusion. ALP is identified as an independent risk factor for MBS, suggesting that significantly elevated ALP levels should prompt consideration of MBS as a likely diagnosis. This finding underscores the importance of ALP as a diagnostic marker in distinguishing between malignant and benign causes of biliary obstruction.

The latest guidelines from the American Gastroenterological Association (AGA) emphasize that in the evaluation of biliary strictures, the primary consideration should be safe, accurate, and convenient diagnosis (1). A meta-analysis, encompassing 11 studies with a total of 356 patients, found that 54% (193 patients) initially diagnosed with biliary strictures via endoscopic biopsy were, in fact, suffering from malignant tumors (25). The complexity of the biliary structure and the narrowness of its lumen pose challenges in obtaining direct histological evidence, even when the stricture location is identified (26). Pathologies in organs adjacent to the biliary tract, such as the liver and pancreas, can impact the bile duct’s patency, further complicating diagnosis. Our study included a 12-month follow-up to confirm MBS diagnoses. As a result, repeat examinations for high-risk patients are essential, challenging the clinical physician’s disease understanding and decision-making accuracy. Past patient screening methods relied on subjective judgments, often overlooking patients with milder clinical signs, since not all biliary stricture patients undergo pathological examination. Our model revealed characteristics of biliary strictures caused by malignancies as exhibited in laboratory tests and ERCP examinations, identifying their risk factors. It enhances doctors’ and patients’ comprehension and interpretation of risk assessment results. The model’s intuitive display facilitates effective doctor-patient communication, supporting personalized diagnosis and treatment strategies.

This study has several limitations. Firstly, as a dual-center study, potential biases may exist. Future efforts will incorporate data from additional centers to enhance the predictive model’s effectiveness. Moreover, the limited sample size of this study restricts the number of independent variables and the variety of machine learning models employed. Finally, as a retrospective study it was difficult to collect enough high-quality images for direct image analysis, only some key endoscopic features could be extracted.Further prospective research will involve a broader range of variables, including clear endoscopic images and laboratory indicators after ERCP drainage, in order to enrich the study.

## 5 Conclusion

Our study successfully leveraged patients’ medical histories, laboratory test results, and specific examination markers to construct a highly efficient predictive model. This model provides robust data-driven support for clinical decision-making processes, facilitating the accurate identification of patients at high risk for MBS. It aids in the formulation of diagnostic and treatment plans, thereby improving patient outcomes.

## Data availability statement

The datasets presented in this study can be found in online repositories. The names of the repository/repositories and accession number(s) can be found below: https://www.jianguoyun.com/p/DUjDqowQ1IO9DBj4o7oFIAA.

## Ethics statement

The studies involving humans were approved by the Ethics Committee of the First People’s Hospital Affiliated with Jiangsu University (Approval Number: K-20220105-Y). The studies were conducted in accordance with the local legislation and institutional requirements. The participants provided their written informed consent to participate in this study. Written informed consent was obtained from the individual(s) for the publication of any potentially identifiable images or data included in this article.

## Author contributions

QY: Conceptualization, Data curation, Investigation, Methodology, Project administration, Software, Validation, Visualization, Writing – original draft. LN: Conceptualization, Data curation, Investigation, Methodology, Project administration, Software, Validation, Visualization, Writing – original draft. JX: Conceptualization, Data curation, Investigation, Methodology, Software, Validation, Visualization, Writing – original draft. HL: Writing – original draft. XZ: Conceptualization, Formal analysis, Methodology, Project administration, Validation, Writing – original draft. MW: Conceptualization, Formal Analysis, Project administration, Supervision, Writing – review & editing. JY: Conceptualization, Data curation, Formal analysis, Funding acquisition, Project administration, Supervision, Writing – review & editing.
